# Iron Deficiency Anemia in Pregnancy after Bariatric Surgery: Etiology, Risk Factors, and How to Manage It

**DOI:** 10.1055/s-0043-1776026

**Published:** 2023-11-09

**Authors:** Carolina de Freitas Alves Amaral-Moreira, Guilherme Redezuk, Belmiro Gonçalves Pereira, Anderson Borovac-Pinheiro, Patricia Moretti Rehder

**Affiliations:** 1 Departamento de Tocoginecologia da Faculdade de Ciências Médicas, Universidade Estadual de Campinas, Campinas, SP, Brazil

**Keywords:** Bariatric surgery, Anemia in pregnancy, Anemia after bariatric surgery, Cirurgia bariátrica, Anemia na gestação, Anemia após cirurgia bariátrica

## Abstract

**Objective** Pregnancy after bariatric surgery is a reality of the 21 ^st^ century and therefore is essential that all obstetricians know how to manage it. The most prevalent nutritional deficiency is iron deficiency and, consequently, anemia. Although bariatric surgery and pregnancy are already risk factors for anemia, we evaluated in our study if there were any other risk factors and actions to improve hemoglobin levels in this population.

**Methods** We performed a retrospective cohort study, and performed frequency measurements and analyzes of odds ratio, X ^2^ and Fisher exact test to evaluate the risk factors.

**Results** We evaluated 44 pregnancies after bariatric surgery, with an incidence of anemia of 62%, and the only identifiable risk factor for anemia was being black. As for the treatment, the iron salt used for oral supplementation did not associate with anemia risk, and in 27% of the patients, the adjustment of the oral dosage was enough for improvement in hemoglobin levels, but in 36% supplementation with intravenous iron was necessary.

**Conclusion** Being black is a risk factor for anemia. The type of iron salt does not correlate with the incidence of anemia, and for the treatment and improvement of iron dosages, it seems an effective increase in iron intake.

## Resumo

**Objetivo** A gestação após cirurgia bariátrica é uma realidade do século XXI e, portanto, é de suma importância que os obstetras saibam conduzir o pré-natal dessas gestantes. A deficiência nutricional mais prevalente nessa população é a deficiência de ferro, que tem como consequência a anemia. Apesar da própria gestação e da cirurgia serem fatores de risco para anemia ferropriva, realizamos um estudo para avaliar se existem outros fatores que são de risco e quais condutas podem melhorar os níveis de hemoglobina nessa população.

**Métodos** Trata-se de um estudo de coorte retrospectiva, e foram realizadas medidas de frequência e análise odds ratio, X ^2^ , e teste de exato de Fisher para a avaliação dos fatores de risco.

**Resultados** Foram avaliadas 44 gestações após cirurgia bariátrica com incidência de anemia de 62%, sendo que o único fator de risco identificado foi a etnia preta. O sal de ferro utilizado na reposição não se associou com o risco de anemia. Em somente 27% das gestantes o ajuste da dose oral de ferro foi suficiente para corrigir a anemia, enquanto em 36% foi necessária a suplementação com ferro endovenoso.

**Conclusão** Ser de etnia preta foi fator de risco para anemia após cirurgia bariátrica e o tipo de sal de ferro para suplementação não se correlacionou com a incidência de anemia. Para o tratamento da anemia, somente o ajuste da dose da medicação parece ser suficiente para a resolução desta.

## Introduction

Pregnancy after bariatric surgery is a 21 ^st^ century reality. Although there is still a lack of studies that investigate the prevalence of pregnancy after this surgery, it is well established that the main operated population are women in reproductive age in Brazil and worldwide. Consequently, obstetricians all over the world must know how to assist these women, understanding the risks and possible complications. 1 2

The most prevalent side effect after bariatric surgery is anemia. Although there are different surgical techniques, all of them are risk factors for anemia. The reduction of gastric fundus decreases chloride acid production, essential for the acid environment necessary for iron absorption. Moreover, there is a decrease of intrinsic factor, responsible for vitamin B12 absorption. Therefore, these women are at risk for both iron and B12 deficiencies. This problem can be intensified during pregnancy, since it increases iron and vitamin B12 consumption, as well as physiologic hemodilution, make pregnancy itself a risk factor for anemia, which is worsened after bariatric surgery. 2 3

Besides those factors, anemia is still a health problem in Brazil, with a prevalence of almost 25% in women of childbearing age, as shown by a study conducted by the government in 2006. Although data from 2013 and 2014 has shown lower rates of anemia in this population, this index in 2013 was still close to 12%. This risk also surges if the woman is black, of low education, or resident of the North and Northeast regions of the country. 4

Anemia in pregnancy is deleterious both for the fetus and for the mother since it increases the risk of prematurity, low birthweight, infections, and postpartum hemorrhage. Severe anemia even increased fetal mortality rates, and at long term, behavioral changes. 5 6

The objective of the present study was to evaluate prevalence, type of anemia, risk factors of severe anemia, need for intravenous iron, oral iron therapy, and perinatal results among pregnant women after bariatric surgery in a university hospital.

## Methods

We performed a retrospective cohort study in the Women's Hospital Centro de Atenção Integral da Saúde da Mulher José Aristodemo Pinotti from the University of Campinas. The study was approved by the institution ethical committee under CAAE 29661920.7.0000.5404.

The study included all pregnant women with previous bariatric surgery with singleton pregnancies followed in our prenatal clinic from January 2015 to December 2020. Women who lost antenatal follow-up were excluded from the study. All data was extracted from the electronic medical charts of the patients and included age, ethnicity, marital status, previous pregnancies, type of surgery, interval between surgery and pregnancy, weight before pregnancy, weight gain during pregnancy, medication used and dosage, incidence of gastric symptoms such as nausea and vomiting, acceptance of oral medication, incidence of gestational hypertension and diabetes and need for medical treatment, need for intravenous iron, hemogram, ferritin, iron and B12 dosages during pregnancy (one each trimester), smoking status, gestational age at labor, labor, ultrasound exams (fetus weight, amniotic fluid index, Doppler assessment) birthweight, breastfeeding, and contraception method after birth.

Anemia was defined by hemoglobin levels < 11 g/dL in the 1 ^st^ and 3 ^rd^ trimesters and < 10.5 g/dL in the second. Hemoglobin levels > 10g/dl were considered mild anemia, 9 to 10 g/dL was considered moderate anemia, and hemoglobin levels < than 9g/ dL were considered severe anemia. Iron deficiency was defined by levels < 50ug/ dL, ferritin deficiency by levels < 13 ng/dL and B12 < 197 pg/dL. Anemia was also classified as normocytic (mean corpuscular volume [MCV] of 82 to 98 fL) microcystic (MCV < 82 fL) or macrocystic (MCV > 98 fL) and normochromic (mean hemoglobin per red blood cell [MCH] between 27 and 32 pg), hypochromic (< 27 pg) or hyperchromic (> 32 pg). We also analyzed which iron salt patients were taking, since new formulations such as ferric citrate might be more tolerable than iron sulfate, and corelated it with the incidence of anemia. 7 8 9

Adequate weight gain was defined by initial pregnancy body mass index (BMI). Patients with a BMI < 18.5 kg/m ^2^ should gain between 12.5 to 18 kg; those with a BMI between 18.5 and 24.9 kg/m ^2^ should gain between 11.5 and 16 kg; those with a BMI between 25 and 29.9 kg/m ^2^ should gain 7 to 11,5 kg; and those with a BMI > 30 kg/m ^2^ should gain 5 to 9 kg. 10

To analyze the data, we used frequency measures, as incidence and prevalence and to corelate date we used measures of chi-squared and Fischer exact test with a significance lever of *p* < 0.05. The software used to perform statistical analyzes of the data was the Statistical analyzes system version 9.2. 11

## Results

We identified 45 patients with 46 pregnancies. One woman had twin pregnancy and another one had an abortion in the 1 ^st^ trimester (both were excluded). The total analyzed cases were 44 pregnancies. Most patients in our sample were married (70%), white (80%), > 30 years old (70%), nonsmokers and did not consume alcohol. In our sample, the type of surgery was described in 27% of the patients, and of them, most were submitted to gastric bypass (58%), the others variated to sleeve or gastric band, and the procedure type did not correlate with anemia incidence. A total of 27% of the patients still had a body mass index (BMI) before pregnancy > 35. The incidence of anemia in our sample was 61% of the patients, 38% of the patients in the 2 ^nd^ trimester and of 56% in the 3 ^rd^ trimester. The most common anemia type was microcystic and hypochromic anemia (56%), and iron deficiency was present in most patients with it (72%). Low ferritin levels were also associated with a higher risk of anemia ( Table 1 ).

**Table 1:** Type and frequency of anemia in pregnant women after bariatric surgery in all trimesters

Anemia ( *n* = 27)	Frequency	Percentage
**Microcystic and hypocrhomic**	15	56%
**Normocistyc and normocrhomic**	12	44%
**Macrocystic and hyperchromic**	0	0

We correlated the incidence of anemia with the type of iron patients consumed. Most patients consumed iron sulfate, alone or associated with other vitamin supplements. There was no statistically significant difference between the type of iron consumed with the incidence of anemia ( Table 2 ).

**Table 2:** Iron salt used by the patients during pregnancy and its correlation with the incidence of anemia

Iron salt type ( *n* = 43)	Anemia	No anemia	Total
I **ron sulfate**	7	5	12
I **ron glycinate**	1	1	2
I **ron fumarate**	6	4	10
I **ron polymaltose**	2	1	2
I **ron carbonate**	1	0	1
I **ron fumarate + iron sulfate**	8	5	13
I **ron sulfate ** + i **ron glycinate**	2	0	2

Fisher exact test: *p* = 0.983.

Severe anemia was rare in our sample, with an incidence of 2% in the 2 ^nd^ trimester and of 5% in the 3 ^rd^ . The most common was mild anemia (Hb levels > 10g/dL) in both the 2 ^nd^ and in the 3 ^rd^ trimester. We also evaluated the evolution of hemoglobin levels from the 2 ^nd^ to the 3 ^rd^ trimester in patients who presented anemia in the 2 ^nd^ trimester. A total of 34% of the patients with anemia progressed with lower hemoglobin levels. Intravenous iron was prescribed to 59% of the patients who presented anemia in pregnancy for hemoglobin improvement. In the patients that had an increase in their hemoglobin levels, 36% of them had intravenous iron prescribed, while in 27% the prescription of higher dosages of oral iron was effective. In 13% of the patients there was an association of intravenous iron and improvement of oral iron dosages, and in 13% the polivitamin was associated with another iron supplementation. Macrocytic anemia was not detected in this sample. Of other deficient nutrients, vitamin B12 deficiency had an incidence of 19%. The only factor that was associated with anemia besides iron and ferritin levels was skin color, since being black was a risk factor for it. Weight gain during pregnancy, BMI before pregnancy, and interval between surgery and pregnancy were not correlated with the prevalence of anemia in our study ( Table 3 ).

**Table 3:** Variables

Risk factor	Anemia	Non anemia	
Interval between surgery and pregnancy			Fisher exact test *p* = 0.6
< 1 year	0	1	
1 to 5 years	11	6	
> 5 years	15	10	
Ethnic group			*p* = 0.026
White	18	17	
Black	9	0	
Body Mass index (BMI)			Fisher exact test *p* = 0.41
< 25	5	3	
25–30	11	3	
30–35	6	4	
35–40	4	5	
> 40	1	2	
Weight gain during pregnancy			X ^2^ = 0.99 GL = 2 *p* = 0.608
Adequate	12	5	
Inadequate (less than stipulated)	5	4	
Inadequate (More than stipulated)	10	8	

The incidence of gestational hypertension or chronic hypertension in this sample was of 13%, and of preeclampsia, 2%. None of the patients required > 1,5 g of methyldopa per day for blood pressure control. Diabetes prevalence was of 25%, and of these, 81% were gestational diabetes. A total of 72% of the patients with diabetes had an adequate treatment with diet only. Diabetes and hypertension did not have statical association with anemia. Regarding fetus development, no major fetal malformations were detected in our study. Considering aneuploids, one patient had a baby with down syndrome, in the anemia group. In our study, newborns from both groups had similar mean birthweight, gestational age at birth, height, and Apgar scores of the 1 ^st^ and 5 ^th^ minutes. In both groups, cesarean was the most prevalent delivery form, and the main indication was previous cesarean deliveries ( Table 4 ).

**Table 4:** Comparison between anemia and no anemia group and neonatal outcomes

Anemia	Yes ( *n* = 27)			No ( *n* = 17)			Mann-Whitney test for comparison between two groups
	mean	min	max	mean	min	Max	
Fetal weight (g)	2977	2665	3620	2975	1940	3930	*p* = 0.95
1 ^st^ minute APGAR	8.88	7	10	8.69	6	10	*p* =0.72
5 ^th^ minute APGAR	9.68	8	10	9.63	9	10	*p* = 0.48
Height (cm)	47.90	43	51	47.69	45.5	51.5	*p* = 0.41
Gestational age (weeks)	38.85	36	41	38	36	40	*p* = 0.33

Sixty percent of our patients returned to our ambulatory after labor, and of them, 62% were exclusively breastfeeding. A total of 37% had tubal ligation during labor, and 40% of them chose trimestral injections as contraception and 10% intra uterine device (cooper or levonorgestrel).

## Discussion

In the present study, we analyzed the prevalence of anemia in pregnant women after bariatric surgery, and the other risk factors associated with it, as the use of different iron salts and what was effective to improve hemoglobin rates. Although some studies have presented weight gain, and an interval between pregnancy and surgery as risk factor for anemia in pregnancy after bariatric surgery, our study has not corroborated such findings, but showed that black women are under a higher risk of anemia.

The incidence of anemia in our sample was of 61%, which is similar to that of other studies that evaluated the incidence of anemia in pregnancy after bariatric surgery. This rate, as expected, is higher than the prevalence of anemia in pregnant women in general, that is, near 42%, since bariatric surgery itself is a risk factor for anemia. 10 12

Our population was divided into gastric bypass and restrictive procedures. Even though is stipulated that malabsorptive procedures, such as gastric bypass, might be associated with a higher risk of anemia than restrictive procedures, both malabsorptive and restrictive procedures reduce the gastric fundus, responsible for the acid pH necessary for iron absorption; therefore, all procedures might predispose to anemia and that is perhaps why procedure type was not relevant in our sample. Another reason for the type of procedure do not associate with anemia was a limitation of our study that not all patients had their type of surgery described in the medical charts. Although anemia is also associated with a short interval between surgery and pregnancy, it was also not significant in our study since, except for one, all patients had at least 1 year of interval between pregnancy and surgery. 10

Our study, different from others that evaluated anemia in pregnant women after surgery, analyzed if the type of iron salt has any correlation with the incidence of anemia in this population. It has been considered that some iron salts might be more tolerable than others, since some formulations like iron sulfate may predispose to gastric symptoms. As far as we are concerned, no previous study had made such analyzes in this population. Our data demonstrated that the type of iron salt does not correlate with the incidence of anemia in this specific population. Although women after bariatric surgery had not been previously analyzed, it has been demonstrated that all iron salts seem effective to prevent and treat iron deficiency after bariatric surgery and iron deficiency in pregnancy, and, as concluded by our study, iron deficiency in pregnant women after bariatric surgery. 8 10 13 14 15

Like other studies, vitamin B12 and folate anemia were not relevant. For folic acid, this is a result of mainly two factors: first, Brazilian food is enhanced with folate, which decreases the necessity of folic acid, and that pregnant women are advised to take 1 pill of 5 mg daily of folic acid, which is a much higher dosage than required daily. As for vitamin B12, most patients were advised by the gastric surgeon to take vitamin B12 supplementation, especially intramuscular, which might justify the low incidence of anemia. 13

As a new finding, our study demonstrated that black pregnant women submitted to bariatric surgery had a higher risk of developing anemia in pregnancy than white pregnant women. That is coherent with government data that demonstrated that being black is a risk factor for anemia in the general population, so it is logical that this applies to pregnant women as well. What raises the question is why: are we not providing the same treatment and assistance according to ethnicity as shown in other studies? Or the access to treatment and healthcare is limited due to social and economic conditions? None of those women had history of sickle diseases or of any other that might justify this prevalence of 100% of anemia. Since these women are at higher risk, we must analyze economic factors and access to medications and care, specially of those patients. 4 16 17

Although iron deficiency anemia is correlated with low birthweight and preterm birth, in our sample that did not occur, even though bariatric surgery is also a risk for such developments. Major malformations were not detected either and we only had one case of aneuploidy (down syndrome), which is more likely to be associated with maternal age than with the bariatric surgery, since an association of increased risk of aneuploidy after surgery is not described in the literature. 10 13

Considering the postnatal follow-up, 62% of our patients were exclusively breastfeeding their babies after 40 days, which is unsatisfactory considering that the World Health Organization (WHO) stipulates that exclusive breastfeeding should be maintained up to 6 months old. On the other hand, this is a good result if compared with another study performed in Brazil, in which this rate was near 40%, and it is close to the European rate of 67%. Maternal milk after bariatric surgery is as adequate for the baby as any other women's milk, and therefore we should stimulate considering the benefits for mother and baby like immune protection and nutrition, among others. 18 19

In the postnatal follow-up, the preferred method was trimestral injection, which is not the method of choice in these patients, due to the weight gain. Most patients after surgery tend not to tolerate this adverse effect and therefore have low adherence to it. The best methods would be long action reversible contraception, especially levonorgestrel intrauterine devices or etonogestrel subdermal implants, since they decrease menstrual bleeding, in a predisposed population for anemia. Unfortunately, subdermal implants are not available in our service. 10

Our study had some limitations. First, it is a small sample, even though it was possible to obtain statical significance from our data. Second, since this was a retrospective study analyzing medical charts, some data was missing, like type of surgery, which prevents us from making further analyzes. Our hospital is an exclusively obstetrical and gynecology hospital; therefore, we do not have access to their surgery report, and most patients do not know the procedure they were submitted to, which might explain the lack of information. Another limitation of the present study is that we did not have the dosages of elemental iron bioavailability for more accurate data.

In another perspective, our study innovates presenting that all iron salts seem effective in the iron deficiency treatment, and more relevant that changes of the iron salt, it, is improving iron intake. Intravenous treatment is also important and effective, and eventually the only alternative for patients that present many gastric symptoms, which is common in pregnant women and after bariatric surgery. 10

Our study also innovates demonstrating that ethnicity is also a risk factor for anemia. Studies have showed that ethnicity has an impact in medical treatments and health developments and therefore we need to provide better care for black women. 16 17

## Conclusion

Anemia and iron deficiency are still an important health issue, especially in pregnant women after bariatric surgery. In our sample, the only other risk factor for anemia was being black. To manage it, proper screening and prescription of optimal dosages of iron is essential. As reported, all iron salts seem appropriate for these women.
